# The ‘slow‐burn effect’ of human trafficking following disaster

**DOI:** 10.1111/disa.12685

**Published:** 2025-04-29

**Authors:** Chris Weeks

**Affiliations:** ^1^ SOAS University of London United Kingdom

**Keywords:** human trafficking, internal displacement, migration, Philippines, risk, Southeast Asia, vulnerability

## Abstract

A disaster is frequently cited as a driver of human trafficking, with claims that earthquakes, tsunamis, or typhoons create a chaotic post‐calamity environment ripe for traffickers to recruit their victims. Theory suggests that increased poverty, displacement, and a breakdown of law and order contribute to this situation. Yet, there is little discussion in the literature of how post‐disaster trafficking unfolds, coupled with a dearth of empirical evidence. This paper challenges existing disaster–trafficking assumptions through interviews with trafficking survivors in the Philippines, a disaster‐prone nation and an averred ‘trafficking hotspot’. Interviewees indicated that disaster‐related disruption to their lives prompted a chain of events which resulted in trafficking many years later—in other words, a notable ‘slow‐burn effect’. These are presented here as five disaster–trafficking narratives or themes, which paint a more nuanced picture than the oft‐held assumption that traffickers exploit people directly in a disaster zone, in the immediate aftermath of the event.

## 1 INTRODUCTION

Claims that disasters increase the risk of human trafficking have become ‘an integral part of the disaster narrative’ (Montgomery, 2011, p. 395), including news headlines such as: ‘Criminals may be trafficking orphans’ (Aglionby, Steele, and Whitaker, 2005); ‘Indian gangs found trafficking women from earthquake‐hit Nepal’ (Burke, 2015); ‘Traffickers targeting Haiti's children’ (Evans, 2010); and ‘Human trafficking prevalent in post‐typhoon Philippines’ (Santos, 2014). The disaster–trafficking discourse leans on a narrative that vulnerabilities are acutely heightened in the aftermath of an earthquake, tsunami, or typhoon, plunging those affected into a state of economic desperation that elevates their risk of exploitation. In the mind's eye, there may be a picture of a chaotic post‐disaster scene with decimated communities and traffickers lurking on the sidelines as law and order breaks down.

On paper, therefore, the disaster–trafficking theory is a compelling one, with academics citing numerous trafficking drivers that are augmented in the post‐disaster environment, such as poverty, displacement, and forced migration (Aronowitz, 2009, p. 23; Shelley, 2010, p. 53; Goździak and Walter, 2014, p. 58; UNODC, 2021, p. 9). Yet, despite the continuous news headlines, reference as fact to disaster–trafficking in non‐governmental organisation (NGO) reports, and many offerings of academic theory, scholars note that the link has never been empirically investigated or proven in the literature (Goździak and Walter, 2014, p. 58; Bowersox, 2018, p. 5; Gurung and Clark, 2018, p. 303). This is against the backdrop of concerns about the state of empirical trafficking research more generally (Zhang, 2009, p. 178; Mahmoud and Trebesch, 2010; Weitzer, 2015, p. 224; Okech et al., 2018, p. 109).

This study examines the disaster–trafficking nexus and begins to fill this void of evidence with testimony from 33 trafficking survivors in the Philippines. These individuals, based at three trafficking shelters in the disaster‐prone Central Visayas region, had all experienced human trafficking as defined by the Palermo Protocol^1^ of 2000; they were invited through a series of interviews to explore the chain of events leading up to their trafficking experiences. In cases where respondents cited disaster as a distinct factor in the run‐up to the act of human trafficking, their perception of how this may have increased their vulnerability is assessed.

## 2 LITERATURE REVIEW

There is a small but growing volume of literature on the nexus between disaster and trafficking, which, to my knowledge, is all theoretical research. The first time the disaster–trafficking connection was made appears to have been in relation to the Indian Ocean tsunami on 26 December 2004, claiming around 230,000 lives in 14 countries (U.S. Geological Survey, 2004). Widespread claims of child trafficking surfaced almost immediately (Aglionby, Steele, and Whitaker, 2005; Nishiyama, 2005; U.S. Department of State, 2005); consequently, ‘various stakeholders, including civil society, academics and organizations, began to conduct awareness‐raising measures in areas at risk of human trafficking’ (Coelho et al., 2016, p. 6). Another major disaster which spurred such claims was the Haiti earthquake on 12 January 2010 (Delva, 2010; Cohen, 2018), specifically with respect to the trafficking of children for adoption (UNDRR, 2012; see also Aglionby, Steele, and Whitaker, 2005). And there are numerous other examples, including Typhoon Haiyan/Yolanda, which struck the Philippines in 2013 (Branigan, 2013; IOM, 2014; Santos, 2014), and the Nepal earthquake of 2015 (Burke, 2015; Childs, 2016).

### 2.1 The disaster–trafficking nexus in literature

The disaster–trafficking claims made by NGOs and government officials, echoed in the media, have been explored in the literature, with theoretical explanations dating back more than 15 years. For instance, it has been posited that recruitment for trafficking is more likely to happen during economic crises, disasters, and conflicts because ‘there is a ready supply of potential victims’ (Shelley, 2010, p. 94). Furthermore, during a disaster, the ‘supply of available children increases, the demand for those children increases, and the intentions of those transporting the children is difficult, if not impossible, to assess’ (Atzet, 2010, p. 510).

Movement in the wake of disaster has been cited as a common risk factor, as post‐calamity conditions ‘force people to seek work far from home or to migrate to survive’ (Sigmon, 2008, p. 246). It is noted that, without savings, sometimes lost due to natural hazards, an education, or advanced skills, and with limited access to formal employment, ‘these migrants have minimal bargaining power to assert their rights and can become easy targets for exploitation’ (Coelho et al., 2016, p. 4).

The link between smuggling and trafficking is also highlighted in disaster–trafficking research, with observations that individuals in a humanitarian crisis ‘in physical danger or dire economic situations might be actively seeking out smugglers to facilitate their migration from crisis‐affected areas’ (Goździak and Walter, 2014, p. 58). Smuggling increases the risk of trafficking because individuals can lose agency during the process and become controlled by the smugglers (Tamura, 2011, p. 2), thus meeting the definition of trafficking. It is often during the transit phase that victims realise something is untoward. They may be held in safe houses and their documents confiscated (Busch‐Armendariz, Nsonwu, and Cook Heffron, 2009, p. 7), or unexpectedly handed over to another employer (Fitzgibbon, 2003, p. 85). In other cases, it is not until they reach the final destination that documents are confiscated, wages do not materialise, freedom is restricted, debts to the employer mysteriously arise, and the job turns out to be something entirely different, such as being forced into prostitution (UNODC, 2013, p. 24).

Alongside rapid‐onset disasters, climate change has more generally been attributed to an increased risk of human trafficking (Molinari, 2017, p. 50). Rising sea levels and drought ‘act to exacerbate [the] existing vulnerabilities and inequalities of local populations that may render them susceptible to trafficking and exploitative labour practices’ (Brown et al., 2021, p. 201); in addition, dwindling fish stocks can force those who work in the fishing industry into a state of economic desperation. Drought combined with conflict, in places where there is no alternative source of income, particularly heightens trafficking risks (Malinowski and Schulze, 2019, p. 143).

Disaster inevitably sits alongside many other social and economic factors that render people vulnerable to human trafficking in the first place; it can be the final ‘nudge’ that pushes individuals into a situation where they face trafficking. The United Nations (UN) and professionals working in disaster risk reduction are at pains to point out that there is no such thing as a ‘natural’ disaster, because a disaster is the effect of a natural hazard that, to some extent, can be controlled or mitigated—for example, by lessening the impact of flooding through the construction of stronger buildings, the erection of flood barriers, timely evacuations, or avoiding building in at‐risk areas. Boyce's (2002, p. 85) theory on ‘mutually‐reinforcing causes for people's vulnerabilities’ highlights the unequal distribution of land; as a result, people head to cities in search of work, where they live ‘in poorly‐constructed squatter settlements on stilts in the water's edge, in low‐lying flood plains and wastelands, and on steep slopes’ (Blaikie et al., 1994, p. 165). Chambers (1983, p. 112) also refers to the ‘ratchet effect’, whereby each disaster makes a population more vulnerable than the last and, consequently, less able to cope.

### 2.2 State capacity

It is often assumed that traffickers can recruit victims more easily after a disaster because of a breakdown of law and order (Childs, 2016), with speculation that the absence or collapse of a state system after an emergency potentially creates protection vacuums, making it easier for traffickers to exploit vulnerable victims (UNDRR, 2012). Yet, it is also claimed that disaster–trafficking is less likely to take place in countries persistently prone to disaster, which have, over time, developed more state capacity to respond (Bowersox, 2018, p. 12).

Further theories suggest that human trafficking flourishes in the wake of a disaster, ‘when there is lack of QoG’ (quality of government) (Tu, 2018, p. 47), which Tu (2018) specifically measures using four different indicators: the International Country Risk Guide's quality of government; government effectiveness; rule of law; and political corruption. Corruption is said to be a significant exacerbating factor in human trafficking (Bales, 2006, p. 88; Guth, 2010; UNODC, 2013), which could arguably increase following the aforementioned breakdown of law and order. Indeed, there is a ‘formula that's working for human traffickers’ in the post‐disaster environment: disrupted infrastructure and overworked law enforcement personnel, coupled with a sudden demand for cheap labour, lead to heightened risks (Hepburn and Simon, 2013, p. 3). Legal frameworks, whether enforced or not, are also brought into question: notably, those who attempted to traffic children following the Haiti earthquake were unable to be prosecuted because of ‘questionable’ trafficking legislation in the United States (Bromfield and Rotabi, 2012, p. 18).

In the case of the Philippines, where my interviews were conducted, it is claimed that there has been significant development of the government's disaster management over the years. ‘[R]apid and comprehensive social welfare reform since 2007’ has resulted in ‘one of the most advanced social protection. .. systems in the East Asia Pacific region, designed to help poor households manage risk and shocks’ (Bowen, 2015, p. 1).

### 2.3 ‘Moral panic’

Given the lack of solid evidence on disaster–trafficking, questions continually emerge over how widespread the phenomenon is, or whether it even takes place at all. It is argued that one motivation for exaggerating or fabricating post‐disaster trafficking is to raise funds for NGOs: generating concerns about the fate of children will shock potential funders, spurring them to donate. It is questioned why ‘this idea, this assumption, always flourish[es] on the heels of a catastrophe’, leading to ‘an emotional investment in the drama of disaster’, with the result of ‘something akin to moral panic’ (Bales, 2021, p. 35).

It is also argued that Western fears about child abuse are ‘globalised and exported’ in disaster contexts so the issue ‘not only becomes more urgent and universal, it also allows fears of child abuse to be discussed, reinforced and constantly validated.. .. The repetition of unsubstantiated claims and unconfirmed reports and the lack of detail allow a blank canvas against which fears can be projected and magnified and which give credence to social anxieties back home’ (Montgomery, 2011, p. 406). There are further observations that disaster–trafficking claims are more prevalent in Western media than in the areas in which the disaster has occurred.

It is reported that ‘after the Indian Ocean tsunami, when the media frenzy died down, UNICEF [United Nations Children's Fund] commissioned assessments of media reporting of the disaster which noted that local newspapers in Indonesia and Sri Lanka were very suspicious of stories of child trafficking from the beginning’ (Goździak and Walter, 2014, p. 59). One anthropological study stated: ‘Like the researchers who tried to track down evidence of trafficking in the post‐tsunami months, I cannot conclude that large‐scale child trafficking indeed took place, although based on the published accounts I think it is likely that at least some children have illegally been given up for adoption’ (Samuels, 2015, p. 230). The author goes on to observe that ‘the perpetual uncertainty about the truth of the rumours has inserted alternative, uncertain futures into parents’ narratives of loss' (Samuels, 2015, p. 230).

While doubts abound over the extent to which disaster–trafficking occurs, it is not generally disputed that the phenomenon does take place to some extent; however, this ‘extent’ remains unknown.

### 2.4 The empirical vacuum

To what degree have these widespread disaster–trafficking claims been investigated in the extant literature? After reviewing all available sources, there appears to be no research at all on a disaster–trafficking link which features empirical evidence from trafficking survivors themselves (Goździak and Walter, 2014, p. 58; Bowersox, 2018, p. 5; Gurung and Clark, 2018, p. 303).

Indeed, a recent and thorough literature review on the topic urges researchers to prioritise the collection of primary data, including from disaster and trafficking survivors. It underlines that ‘primary research offers a necessary rich understanding of the conditions of trafficking post‐disasters and allows a more in‐depth assessment of the variations in experiences between vulnerable groups’ (Hoogesteyn et al., 2024, p. 2886). The authors also call for ‘more localised and context‐specific research’, rather than wide‐ranging analyses of cross‐country archival data (Hoogesteyn et al., 2024, p. 2886). Another recent sweep of the disaster–trafficking literature found just five peer‐reviewed articles from 2016–19, none of which contained empirical research (Curbelo, 2021, p. 11).

This study therefore enters the debate with the following research question: *how can trafficking survivors' accounts help to explain the link between disasters and human trafficking?*

## 3 DEFINITIONS AND CAVEATS

Before embarking on any study into the disaster–trafficking nexus, a number of definitions and caveats are required. First, this paper adheres to the Palermo Protocol when referring to ‘human trafficking’, with its key features of recruitment, transportation, transferal, harbouring, or reception of persons for the purpose of exploitation. Although concerns have been raised about the Protocol's limitations and global effectiveness (Limanowska, 2005, p. 12; Seideman, 2015, p. 2), it is a widely‐used definition of human trafficking and the first to recognise the issue as a criminal offence (Siller, 2017, p. 408). Second, pursuing statistics on human trafficking is complicated by the fact that, while some forms of trafficking, such as that of labour, take place in plain sight, most go undetected. Other complicating factors are: data are not shared between countries, or even authorities (IOM, 2015); survivors do not come forward because they are afraid of repercussions, they are ashamed, or they face stigma (Nykaza, 2010, p. 319); aid agencies, governments, and other bodies may distort figures to further their own aims, such as raising funds (Bales, 2021, p. 34); and statistics cannot be usefully compiled or compared because the definition of trafficking is interpreted inconsistently (Tigno, 2012, p. 32). Furthermore, pressure from news outlets to provide numbers on human trafficking means that ‘[o]rganizations feel compelled to supply them [statistics], lending false precision and spurious authority to many reports’ (Feingold, 2010, p. 10).

Nonetheless, it should be recognised at the outset that human trafficking is without doubt a major international business. Annual profits have been estimated at USD 150 billion (ILO, 2014) and nearly 50 million people were believed to be living in modern slavery in 2021 (ILO, 2022), with these figures representing the ‘tip of the iceberg’ (Quirk and Thibos, 2018).

## 4 METHODOLOGY

This work features interviews with trafficking survivors conducted at three trafficking shelters in and around Cebu City in the central Philippines. The research formed part of a wider study on the impact of Typhoon Haiyan/Yolanda in 2013, although the specific set of interviews presented here are not linked directly to that disaster. The objective was to learn more broadly about survivors' first‐hand experiences of trafficking and their perception of disaster as a catalyst in their trafficking journey. The wider study, for a Doctor of Philosophy dissertation, was carried out from 2016–19, and these interviews were conducted over the course of 10 days in June 2019.

### 4.1 The context of the Philippines

The Philippines was chosen as the site to examine the disaster–trafficking nexus because it is a disaster‐prone nation, ranking ninth on the 2020 World Risk Index, and fourth on the long‐term Global Climate Risk Index based on data from 2000–19 (CFE‐DM, 2021), with 565 disasters claiming 70,000 lives and costing the economy USD 23 billion since 1990 (World Bank Group, 2021). The archipelago is situated on the Pacific Ring of Fire and is susceptible to volcanic eruptions, earthquakes, and around 20 typhoons each year, combined with tropical storms, monsoons, floods, and landslides. Over a five‐year period, 93 per cent of Filipinos reported experiencing at least one typhoon (Bollettino et al., 2018, p. 14). The nation is also averred to be a ‘trafficking hotspot’ (U.S. Department of State, 2014; Guilbert, 2018; Commission on Filipinos Overseas, 2019), although this claim must be balanced against warnings that the term ‘hotspot’ is a gross oversimplification because the logistics of trafficking are far more complex and geographically fragmented (Molland, 2012, p. 66). Attempts to collate trafficking data in the Philippines suggest that, in 2023, 859,000 people were enslaved in the country, representing 0.78 per cent of the population (Walk Free, 2023). The Philippines is recognised as ‘an important case study for understanding community resilience and vulnerabilities, as well as the intersection between poverty and hazards’ (Daly, 2016, p. 10).

## 5 THE INTERVIEW PROCESS

The interviews with formerly trafficked people were semi‐structured and involved a set of questions to ascertain how disaster, ranging from low‐level repetitive environmental hazards to major rapid‐onset events, may have been a direct or indirect catalyst in each respondent's trafficking experience. Disaster included a subset of human‐origin hazards such as fire and demolition, which were cited by a number of respondents, while noting that the line between human‐caused and natural hazards leading to disaster is blurred (Communications Staff, Washington State University, 2006). Overall, each respondent was invited to provide 10 answers to narrative questions and 48 items of quantitative data, including age, place, type of housing, and category of weather event, plus another 18 items of data for each geographical location. Other variables were captured including whether a dwelling was situated in an informal settlement, amenities such as clean water and electricity, and family situation, such as with whom the respondent was living, and any abuse or major incidents in their life at that time. These paint a picture of each individual's resilience to the shock of disaster and enable common themes to be examined. Each respondent was also asked whether they felt that they were forced to move to a new location or did so freely, and whether they were experiencing exploitation or abuse at the time—all of which are key features of trafficking. Where disaster–trafficking specifically emerged as a theme, subsequent in‐depth interviews were carried out with these individuals to garner more details.

As noted, the interviews were conducted at a trafficking shelter with three sites in and around Cebu City that are supporting and empowering people at various stages of post‐trafficking rehabilitation. These comprised: a safe house supporting people soon after trafficking; a drop‐in centre which equipped people with livelihood skills as part of the latter stage of the rehabilitation process; and a residential facility situated around an hour outside of Cebu. All three sites were established by nuns, with a number of support staff running them from day to day. The nuns told me, anecdotally, that they knew that many of the people they were supporting had faced trafficking risks due to disaster. They were interested in finding out more about the phenomenon but had been unable to devote any time to the topic, prompting them ultimately to agree to this research request. Naturally, the findings were shared with them.

It is worth pointing out at this stage that securing interviews with people who have experienced trafficking required a good deal of preparatory work and trust‐building, which started with research visits one year earlier, and introductions from contacts such as academics who knew the shelter's managers. A number of other NGOs which provide support to survivors were also approached, but only one organisation was willing and able to provide access within the timescale required. It is not named to ensure that the identities of the trafficking survivors are protected.

### 5.1 Research partners

Conducting in‐depth interviews with 33 trafficking survivors over a period of 10 days necessitated support. While all interviewees spoke fluent English, as is common in the Philippines, many who had lived in the capital, Manila, preferred to speak in Tagalog, while those from Central Visayas opted to communicate in the regional languages of Cebuano and Waray‐Waray.

I was assisted by three facilitators. The first was an academic and trained social worker from a major university in Cebu, with a career spanning several decades at the city's Department of Social Welfare and Development; I had met her two years earlier as part of this research project. The second was a local freelance NGO worker who had carried out work with the trafficking shelter's sites before, and who was known and trusted by the staff. The third was a member of staff at the shelter, which, as stated above, incorporates three sites. The implications of this were considered; it was concluded that the person was impartial as far as this disaster–trafficking research is concerned and would have no reason to steer interviewees in a particular direction.

After careful consideration and consultation with the centre's managers and the social worker, it was decided that I would be present during the interviews and debrief sessions. Additionally, I was able to pose questions directly to the interviewees, while maintaining awareness that researchers ‘carry multiple identities which have the potential to create power dynamics with respondents’ (DoCarmo, 2019, p. 10). The ambiance appeared relaxed, and almost all interviewees seemed to speak freely, confidently, and at length about their experiences in a group setting. In practice, as well as giving answers verbally, most respondents wanted to fill out many of the answers on the questionnaires themselves, rather than them being recorded by the facilitator. This was a welcome development as it meant that the information captured on the paperwork came directly from the respondents.

### 5.2 Ethics and data protection

This research was preceded by a rigorous two‐stage ethics review at SOAS University of London, United Kingdom. All interviewees were aged 18 or over. Numerous preparatory meetings were held with the shelter's managers to agree on the questions, making sure that they were appropriate and would not create upset or cause trafficking survivors to relive trauma. The presence of the trained social worker, also acting as the lead local researcher, was important. She, alongside trained staff from the trafficking shelter, was on hand to provide support if any respondents required it post interview. Group debrief sessions were held after all of the interviews to ‘check in’ on participants, and to ensure that they were content and had not experienced any emotional challenges which required follow‐up in the supportive environment of the trafficking shelter.

Individual interviews were not audio‐recorded, as they were conducted with facilitators in three simultaneous breakout groups; however, the debrief sessions were audio‐recorded with participants' permission, along with the in‐depth follow‐up interviews. These were captured on a mobile telephone and transferred subsequently to a virtual password‐protected drive—the original file was deleted from the phone. Notes and transcripts were scanned and stored securely in the same way. All names of people who had experienced trafficking have been changed to protect their identity; they were chosen by the respondents themselves, which also served as an ‘ice‐breaker’ exercise at the start of the interview process (UNIAP, 2008, p. 21).

As compensation for interviewees' time, and in consultation with the trafficking shelter, each respondent was provided with five kilograms of rice and PHP 500 (approximately USD 9). At the drop‐in centre and residential facility, which were supporting people in the latter stages of their rehabilitation, this money was handed directly to the respondents to spend as they saw fit. In the case of the safe house supporting people at the earliest stage, many of whom had reportedly been dependent on drugs, this money was withheld by staff until the person moved on to the second stage of rehabilitation. No payments were made to the organisation running the trafficking shelter's sites.

It was made clear from the outset that participants could withdraw from the research at any time, without the need to give a reason, and without a penalty.

### 5.3 Selecting interviewees

At the time of my research visits, 33 people were present and willing to be interviewed across all three of the trafficking shelter's sites. It was determined that there would be enough time and resources available to interview all of these individuals without the need to introduce a selection process beyond the stipulation that they had experienced human trafficking. The shelter's managers clearly had a sophisticated understanding of the Palermo Protocol, and my interviews independently confirmed that, indeed, all respondents' accounts featured every element required to meet the formal criteria of trafficking. It is also worth noting that domestic Filipino trafficking legislation uses the same definition as the Palermo Protocol.

It is especially important to ensure that survivors' accounts do meet the definition of human trafficking because, as stated earlier, the term is often misused by governments and other entities. There are varying motivations for this. For instance, one scholar has documented how two governments—Argentina and the Dominican Republic—used the term ‘human trafficking’ to cover all forms of migrant labour so, it was argued, they could improve their standing in the US *Trafficking in Persons Report* (Brennan, 2014, p. 108) in the face of potential economic sanctions. Caution is needed therefore when approaching human trafficking research, and it is always necessary to verify independently that an individual has experienced trafficking by examining the unique features of each survivor's story.

At the beginning of this research endeavour, I was aspiring to interview trafficking survivors of all genders who had ended up in a range of trafficking destinations owing to the impact of disaster. In the end, though, all of the respondents identified as female, and all had experienced domestic trafficking, exploitation, and abuse through sex work.

It should be acknowledged at this point that the trafficking literature has tended to focus disproportionately on women rather than men, and disproportionally on sex trafficking (Goździak and Graveline, 2015, p. 16). In reality, ‘contemporary victims of trafficking are more likely to be victims of labour trafficking, forced to serve as child soldiers, or trapped in domestic servitude’ (Shelley, 2010, p. 297). My interviews therefore do little to redress that balance in the literature.

It is also helpful to consider why I was only able, as a researcher, to track down this category of trafficking survivor—in other words, female survivors of human trafficking who were exploited through sex work. It is worth dwelling on the question: where did I initially inquire about interviewing trafficking survivors, and why did I take this approach? Looking back through my notes it is clear that I began querying the possibility of hearing directly from trafficking survivors with almost every interviewee I encountered. These people ranged from government officials to police officers, social workers, court officials (including judges), NGO staff members, and academics. It became clear, early in the process, that speaking directly to trafficking survivors would not be accomplished easily. To make the process manageable, with the necessary support and supervision in place, these individuals would need to be in one location and there would need to be a structure in place to support interviewees who were describing potentially traumatic experiences, with guidance offered by trained personnel who knew the survivors and were overseeing their welfare. Moreover, this was alongside understandable reluctance to allow an ‘outside’ researcher to talk directly to people who had experienced such events.

I did speak with a number of NGOs who supported trafficking survivors of all genders, and who were theoretically willing to provide access. Yet, the reality of the project's constraints—in terms of time and limited financing—had to be recognised, meaning that the interviews featured in this paper were the most realistic way to progress with the research. With more time and resources, I believe it would be possible to track down survivors of all genders who were trafficked to varying destinations, adding further value to the existing literature.

Interviewees' ages and home location/trafficking destination are presented in Table 1, along with whether or not they perceived disaster as a factor in their trafficking experience.

**TABLE 1:** Overall summary of interviewees' ages, their home location/trafficking destination, and whether they perceived disaster as a factor in their trafficking experience.

	Name (pseudonym chosen by each interviewee)	Age at the time of the interview	Age when trafficked	Where: home location –trafficking destination	Disaster cited by interviewee as a factor in trafficking? N = natural hazard; H = human‐made hazard; X = disaster not cited as a factor.	Brief narrative
1	Sheena	37	22	Cebu –Cebu/Iloilo	X	Underlying poverty and drug use cited by respondent as contributory factors.
2	Jonalyn	25	15	Rodriguez – Cebu	N	Flooding seen as a contributory factor in displacement, leading to trafficking.
3	Jen	18	14–16 (unsure)	Cebu – Cebu	X	Constant demolition of homes resulting in employment search.
4	Anabel	19	18	Cebu – Cebu	X	Escaped abusive situation, resulting in human trafficking.
5	Mia	46	19	Mandaue – Cebu	X	Abuse and drug use cited.
6	Yurika	34	15	Leyte – Cebu	N	Displacement due to typhoon. Escape from subsequent abuse.
7	Michelle	25	20	Bogo City – Cebu	H	Displacement due to fire cited as a contributory factor.
8	Mardie	19	15	Cebu – Cebu	N	Needed money to rebuild home after flooding.
9	Engel	19	15	Cebu – Cebu	N	Home damaged by typhoon; needed to raise money for family.
10	Lara	22	15	Manila – Cebu	H	Fire and subsequent demolition cited as a catalyst.
11	Mabel	45	14	Cebu – Cebu	H	Fire and demolition of home seen as a contributory factor.
12	Mary‐Ann	30	20	Toledo City – Toledo City	X	General financial pressures led to employment search.
13	Adelaine	28	13–15 (unsure)	Lanao del Norte – Cebu	H	Search for employment after house destroyed by fire.
14	Andrea	23	21	Bohol – Cebu	N	Earthquake damaged home, resulting in financial desperation.
15	Riya	52	16	Oriental Mindoro – Cebu	X	Demolition led to displacement, cited as a contributory factor.
16	Joy	32	20	Zambales – Cebu and Mandaue	N	Displaced as a child due to volcano. Disaster noted as a peripheral factor in vulnerability to trafficking.
17	Maya	31	13	Bogo – Cebu	N	Pushed into work due to economic desperation. Typhoon was a factor.
18	Rowena	26	16	Cebu – Mandaue	H	Fire led to displacement and escaping abuse.
19	Dulce	27	11	Bogo – Cebu	X	Escaping abusive situation.
20	Keshni	23	18	Manila – Cebu	N	Repeated flooding cited as a contributory factor.
21	Nikki	40	15	Mindanao – Cebu	N	Flooding of rivers disrupted education. Peripheral contributory factor.
22	Rosepete	26	12	Mandaue – Mandaue	X	Demolition cited as a contributory factor.
23	Christina	27	15	Mandaue – Cebu	N	House damaged by a typhoon, prompting employment search and subsequent trafficking.
24	Jhuna	19	16	Cebu – Cebu	N	Employment search due to damage of home by a typhoon.
25	Cristal	22	15	Carcar – Cebu	X	Escaping abuse sparked chain of events leading to trafficking.
26	Inday	20	10	Cebu – Cebu	X	Employment search due to escaping abuse.
27	Mich	19	12	Cebu – Mandaue	X	Escaping abuse.
28	Sofia	19	15	Cebu – Cebu	N	Home destroyed by a typhoon, prompting move to known abusers and subsequent employment search to escape.
29	Sophia	21	12	Cebu – Cebu	H	Fire at family home cited as a contributory factor at a young age.
30	Amaya	19	15	Cebu – Cebu	X	Displacement due to demolition cited as a contributory factor.
31	Tanya	20	12	Minglanilla – Minglanilla	N	Experienced repeated flooding and a major typhoon; both cited as contributory factors.
32	Barbara	19	12	Cebu – Cebu	X	Escaping abuse was primary catalyst.
33	Jan	34	27	Samar – Cebu	N	Frequent flooding, evacuations of home, and typhoon combined to impact education.

**Note:** presented in the order in which the interviews were conducted.

**Source:** author.

The selection of trusted facilitators, and the holding of the interviews in the familiar surroundings of the trafficking shelter's three sites, meant that most interviewees were extremely forthcoming, speaking very openly about their experiences. Despite the serious nature of the topic, there were many moments of unexpected humour. During debrief sessions, participants interacted with each other, building on or disagreeing with each other's points.

After hearing interviewees' stories, choosing a ‘label’ such as trafficking ‘victim’ or ‘survivor’ becomes problematic. While perception of demeanour is subjective, none of the interviewees presented as ‘victims’. Ideally, I would have asked respondents themselves which term they would prefer, if any, and will do so if similar research is conducted in the future. I have therefore tried to avoid using descriptive words or labels wherever possible, opting for ‘survivor’ when a general term is needed.

## 6 INTERVIEW FINDINGS

The first issue to address is how many of the 33 interviewees perceived disaster as a factor that increased vulnerability to human trafficking. Table 1 shows the headline data on this issue, presented in the order in which respondents were interviewed.

**FIGURE 1:**
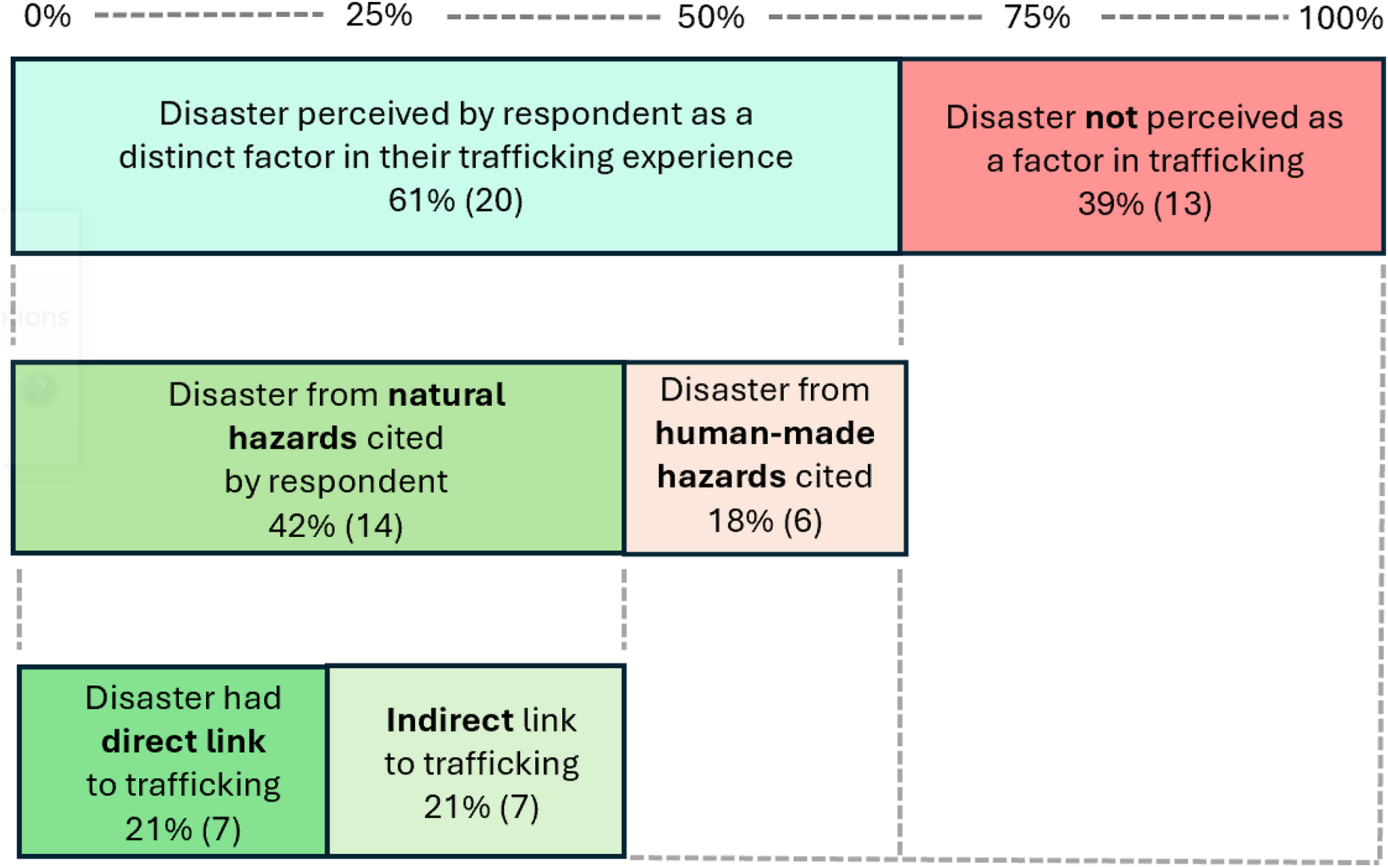
Percentage of interviewees who perceived disaster as a factor in the lead‐up to their trafficking experience. **Source:** author.

It is worth recognising the significant extent to which the interviewees perceived disaster as a driver of trafficking. While extreme care was taken not to ask leading questions, caution is of course also needed to ensure that these aspects of stories are not over‐emphasised. Nonetheless, as stated, the Philippines is a disaster‐prone country so the results may not be surprising. Filipinos accept ‘torrential downpours, typhoons and earthquakes as realities of life’; theirs ‘is a life particularly prone to calamity’, one where ‘they have hardly had time to. .. pick up the pieces before the destruction starts again’ (Bankoff, 2003, p. 162). Figure 1 simplifies the data and shows the proportion of respondents who cited disaster as a factor in their trafficking journey.

It is significant that more than 60 per cent of respondents cited disaster as a catalyst in their human trafficking experience. This is further divided into direct and indirect links. Typically, a direct link is a natural hazard, such as a typhoon, forcing an individual to seek employment due to ensuing financial hardship that subsequently resulted in human trafficking. An indirect link was typically presented as a slow‐onset disaster like annual flooding, adversely affecting family finances, which, over time, prompted a search for employment that resulted in trafficking.

After reviewing trafficking survivors' stories and considering how best to present a large amount of information, I divided the narratives into five themes: economic desperation due to disaster; escaping abuse; eroded parenting standards; migration gone awry; and disrupted education.

### 6.1 Theme 1: economic desperation due to disaster

Poverty, which is known to increase following a disaster (Goździak and Walter, 2014, p. 59), is at the top of the literature list when it comes to trafficking drivers (Aronowitz, 2009, p. 23; Mahdavi, 2011, p. 216). It was no surprise, then, that this study's interviewees frequently referred to economic desperation in their lived experiences. In the Philippines, this risk appears to be magnified by a strong cultural expectation and a desire to support one's family financially. Several respondents stated unequivocally that they would ‘do whatever it takes’ to secure funds for their family, and would risk their own health and even their own life to achieve this.

Facing financial hardship after a disaster, many respondents were therefore clearly ready to take jobs that were ‘too good to be true’ and proceed with off‐the‐books job offers, resulting in recruitment, deception, and exploitation, thus meeting the definition of trafficking. For instance, Engel, 19, was trafficked at the age of 15 after what she describes as a happy childhood. She did not report any abuse during her early years, in contrast to many other respondents. Her family lived in a basic house made of wood, with an iron roof which was damaged during a typhoon, destroying many of their possessions. Engel recalled: ‘We were kept awake, and watched the water coming into our house. I was forced into work because of money problems, so that I could repair the second floor of our home. It is very important to help my family because they are the reason for my existence. I want to repay the sacrifices of my parents’. Andrea, 23, was trafficked at the age of 21 and described how the family home was damaged first by a typhoon, and then by an earthquake. She stated: ‘Because of these events, I needed to have money to buy food, new things, and so on’; a subsequent search for work evolved into human trafficking.

Similarly, Sofia, 19, the youngest of eight siblings, was trafficked at the age of 15. She had been living in a basic house, made of wood with an iron roof, which was damaged beyond repair by a typhoon, prompting the family to build another basic property next to it on the same site. Sofia said: ‘When I was younger, we stayed in one place the whole time, but, during a typhoon, our house was damaged. We had to rebuild our house, so we needed more money to do this. It is very important for me to provide for my family because I want them to be happy’.

Maya, 31, faced two disasters during her childhood, and believes this made her more vulnerable to human trafficking. She explained: ‘Because of the flooding from Typhoon Ruping, we lost our farm and we didn't have enough income to sustain our daily needs. Later, our farm plants were eaten by pests. We lost everything, and never really got it back. Because of these calamities, I was forced to help through earning money. I was recruited by my friend and influenced. I had to help my family: my father was bedridden. My friend took advantage of me and recruited me, and also got me hooked on drugs’. Super Typhoon Ruping, known internationally as Typhoon Mike, occurred in 1990 when Maya was just three years old; however, she underlined that the financial knock‐on effect for her family continued throughout her childhood, resulting in her being recruited into work, and trafficked, at the age of 13.

Christina, 27, the youngest of five siblings, was also trafficked in childhood, at the age of 15. As with other respondents, she lived in a house built of ‘light materials’ that was susceptible to storm damage, with few facilities. Christina said that ‘because of the typhoon, our house started leaning. Because of the poor condition of my house, and inadequate income, I was forced to work in a bar as a dancer. I was deceived by my friend: she told me that we will apply to work as waitresses. But when we arrived there, she said it was already closed. And so she blackmailed me to dance just one night, so that we can have money for going home’.

Another respondent, Jhuna, 19, described how the roof of the family home blew off in a typhoon. She sought work as a 16‐year‐old to pay for repairs and to avoid the threat of the structure being demolished. She was subsequently trafficked during this search for employment.

### 6.2 Theme 2: escaping abuse

The ‘escaping abuse’ scenario portrayed by interviewees is part of a two‐step trafficking driver: disaster forces people to enter into an abusive situation, which leads to them seeking risky employment to escape, which then morphs into trafficking. For example, Rowena, 26, was living with her grandmother who ran a business. An arson attack destroyed this property, which forced her to move back in with her abusive father. She wanted to escape at any cost and took a risk on working with an employer who deceived and exploited her, thereby meeting the definition of human trafficking. The link between the initial disaster and the final outcome of trafficking is therefore apparent in this case, albeit occurring over a significant amount of time (10 years) and with numerous ‘steppingstone’ events leading up to the act itself.

Family breakdowns, including divorce, alcohol abuse, or the death of a parent are already cited in the literature as trafficking risk factors. This can result in people migrating to cities, and increased alcohol abuse and violence, with ‘familial exploitation often becoming a steppingstone to abuse by traffickers’ (Shelley, 2010, p. 53). According to this study's interviewees, abuse can happen because parents or caregivers become frustrated with the situation in which they have been left by a disaster, such as the loss of livelihood, self‐worth, and income, which results in them ‘taking it out’ on members of their own family. This was the case with Yuri, 34, who was forced to move back in with a known abuser owing to weather‐induced displacement. Yuri, who was trafficked at the age of 15, stated: ‘I ran away from home because I was abused by my brother, and my mother didn't believe me. I moved from one place to another because of abuse and I needed to earn money. But I did not have enough education, so I entered into laundry and housemaid work, but was deceived’.

While presenting different narratives, these cases point to the sequence of events unfolding as follows: disaster → abuse → escaping abuse → desperate employment search → trafficking. Once again, it should be emphasised that the passage of time between the disaster and actual trafficking can be long, pointing to a continual ‘slow‐burn effect’.

### 6.3 Theme 3: eroded parenting standards

The third scenario cited by respondents was disaster leading to a change in parental attitudes or standards. This relates to parents or caregivers struggling to cope with the ensuing economic hardship, displacement, or deaths in the family, leading to a sense of despair. Yurika, 19, was trafficked at the age of 12. A fire which devastated the family home ‘added to her parents’ problems', meaning that they were subsequently unable to look after her appropriately. Yurika said: ‘The fire caused my parents' demeanour to change: they lost hope. I felt rejected and betrayed. Because of my young age they forced me to a man, but I ran away from home, and a mother of my friend brought me somewhere and locked me in a house. She sold me to many men’.

Once again, this points to a situation where individuals needed to fend for themselves or to escape an unbearable situation, often with disaster being a contributing element.

### 6.4 Theme 4: migration gone awry

The linkage between migration and human trafficking is widely recognised in the literature, although not specifically in relation to disaster. Human trafficking can be seen as ‘migration gone horribly wrong in our globalised economy’ (Chuang, 2006, p. 138), but it is also observed that trafficking is inextricably associated with migration, during which a continuum of possible abuses may take place (Mahmoud and Trebesch, 2010, p. 175).

Four respondents in my research referred to disaster‐induced migration and displacement as a catalyst for the chain of events which resulted in trafficking. Jonalyn, 25, experienced a ‘life on the move’, citing disaster as a contributing factor in her trafficking journey. Similarly, Lara, 22, was trafficked after her family home was demolished following a fire. She remarked: ‘After this, my grandparents, who brought me up, could not provide for me. I was lured by a friend to take drugs; and since my grandparents cannot provide my needs, I was pushed to look for income and a means to earn money. This is so that I can eat and buy some clothes for myself’.

The answers of these respondents confirm that displacement is a clear risk factor in terms of human trafficking, both directly and indirectly. As with the other themes, however, this is not always an immediate risk and can play out over a long period of time.

### 6.5 Theme 5: disrupted education

In the trafficking literature, ‘low income, poor education, and lack of employment’ are noted as risk factors (Aronowitz, 2009, p. 23). Education is frequently interrupted post disaster, which was certainly the case following Typhoon Haiyan/Yolanda in 2013. This event damaged or destroyed more than 2,500 public schools, leaving 12,400 classrooms in need of repair and thousands of students losing their records (Theirworld, 2015), which are difficult to replace.

The situations presented by respondents in my research relate to the impact of leaving school to seek work and support family members. In many cases, this is driven by disaster. A lack of formal qualifications means that individuals can be forced to seek work which is ‘off the books’ or menial, behind closed doors, and thus entailing a higher risk of trafficking.

Nikki, 40, traces back the problems in her life, and ultimately her trafficking at the age of 15 into forced labour, to a lack of education at a young age while growing up in a rural area. She reported that persistent natural hazards were a contributory factor: ‘I couldn't complete [my] education due to flooding of rivers. I needed to cross two rivers, and couldn't get to school. I really wanted to do this. I was deceived by a trafficker, promised to have a better life in Cebu City and finish my study’.

Joy, 32, faced a volcanic eruption as a child, displacing her family; owing to the financial impact, she was forced to take up work at the age of 13, impacting her education. She lived with her grandparents in Manila from the age of four, who then frequently uprooted and moved to different locations. She stated that she was abused as she went from one place to another, and cites these circumstances as contributory factors in the run‐up to her trafficking experience at the age of 20. Compounding these troubles, an education certificate she obtained was later washed away during a typhoon, affecting her subsequent job search.

Jan, 34, left school at 15 to live on her own and work. She said: ‘We needed money to build another house because our house was damaged by a typhoon and there was no more food to eat or water’. When this regular and stable work dried up, she was forced to resort to sex work, which resulted in trafficking at the age of 27.

The International Organization for Migration (IOM) also underscores that migrants without an education or advanced skills have ‘minimal bargaining power to assert their rights and can become easy targets for exploitation’ (Coelho et al., 2016, p. 4). The testimony presented here adds weight to this argument.

## 7 DISCUSSION: STEERING THE DISASTER–TRAFFICKING NARRATIVE

The previous section presented trafficking survivors' reflections on how disaster may have enhanced their own particular vulnerability to human trafficking; however, the interviewees highlighted many other trafficking risk factors in their narratives. This naturally prompts inquiries into whether disaster was the *main* catalyst for trafficking, with these extra factors exacerbating the situation, or whether disaster was merely an exacerbating factor, and the trafficking was destined to take place anyway, with another central risk factor, or myriad factors, at play. It may seem obvious to state that this is a difficult question to answer because it is impossible to ‘wind back the clock’ and relive the experience without the element of disaster to observe if trafficking would ultimately have taken place in each scenario. Nonetheless, it is possible to explore to what extent disaster was a *likely* trafficking driver, and how prominent a role it played.

### 7.1 Additional trafficking drivers

There was no specific interview question on drug use, although most of the interviewees recalled being addicted to drugs either in the run‐up to their trafficking experience or after they had been recruited. For example, Rowena stated that she ‘moved at the age of 13 to recover from drug addiction’, while Cristal, who was trafficked at the age of 15 by the partner of a family relative recalled, as an eight‐year‐old, witnessing a ‘riot and shooting because of drugs’. Indeed, the fallout from drug abuse emerged as a common theme in many respondents' childhoods, often featuring the erosion of parents' ability to care for their children.

In terms of financial pressures and providing for dependents, slightly less than two‐thirds (21 of 33) of respondents had children themselves, with the average number being 2.6. It was clear, from many respondents' answers, that supporting their children—even if they had since been put in the care of others—was central to their lives. A good deal of other socioeconomic information was also captured during the interviews, including parents' employment status, number of brothers or sisters, whether the respondent was the oldest or youngest sibling, and educational attainment. While the findings are too detailed to present in this paper, this was done to identify themes and patterns which could emerge as potential trafficking risk factors.

### 7.2 Disaster: a central or peripheral risk factor?

It appears that disaster was a central feature of four interviewees' trafficking journeys. Three of these relate to typhoons, and one to an arson attack. Referring back to the testimony in the previous section, Sofia pointed to a distinct disaster event in which the family home was virtually destroyed by a typhoon, which meant that the family needed to build a new one; this pushed them into financial hardship, prompting her desperate search for work, which resulted in human trafficking. The disaster is portrayed by Sofia as a distinct phenomenon that immediately changed the family's financial circumstances—and thus sparked the chain of events which led to trafficking. Christina also cited a typhoon as a sudden‐onset disaster which damaged the family home, forcing her to find extra income, which led to trafficking.

This was also the case with Maya, who said the family's farm was lost due to a typhoon, plunging them into poverty; however, they did continue farming for a period afterwards, but their crops were destroyed by pests. The two factors combined led Maya to search for work, resulting in human trafficking. Disaster is therefore a significant, but not the sole, factor in this instance.

Rowena experienced an arson attack which forced her back into an abusive situation; she sought work to escape the situation, which morphed into trafficking. While this is a two‐step trafficking driver—a human‐made disaster prompts a move into an abusive situation, which, in turn, prompts a desperate search for employment to escape—it seems reasonable to conclude from Rowena's narrative that the fire was the primary catalyst.

In the remaining 16 cases involving disaster, the latter was certainly a contributory factor. Yet, after reviewing all of the interview notes and narratives, it cannot be said with certainty that it was the *only* factor—and the experience of human trafficking may have occurred whether or not the disaster had taken place. For instance, in the testimony offered by Engel and Andrea about a typhoon and earthquake damaging their respective homes and spawning financial hardship, disaster was a contributing factor. But it is uncertain whether this was a sole or central factor in heightening trafficking risks.

In the case of Jhuna, the roof of the family home blew off in a typhoon and she needed to get a new job to pay for repairs to the house, which led to human trafficking. It cannot be concluded from the interview transcript that she sought employment solely to fix the house. In this case, therefore, disaster can only be viewed as a contributory factor.

Yuri stated in her interview that a constant barrage of weather events made the family home unliveable, forcing her to seek employment, which resulted in trafficking. While this contributed to the need for her employment search, it cannot be seen as the only factor. And Jonalyn's family was evacuated twice due to flooding, and she was regularly displaced, leading to poverty and uncertainty in her life. Nonetheless, these natural hazards can also be seen as contributory rather than central factors. Similarly, Yurika described a situation where fire ‘added to her parents’ problems', resulting in a chain of events which led to her searching for work and ultimately being trafficked.

Lastly, the cases of Nikki, Joy, and Jan are based on the premise that their education was disrupted by an ongoing disaster. While this is certainly the case according to their testimony, it cannot be said with certainty that this was the *only* reason they did not complete school and sought employment through a process which carried with it higher trafficking risks. Again, this can only be viewed as one element heightening their vulnerability.

### 7.3 The ‘slow‐burn effect’

Having explored how disaster may have contributed to these survivors' experiences, it can be seen that their testimony points to a significant passage of time between the disaster and the moment of human trafficking, and that numerous ‘steppingstone events’ occurred along the way. For instance, a disaster increases poverty, which causes frustration and abuse in the household. This, in turn, prompts an individual to seek to escape the abusive situation in which they have become trapped through a desperate search for employment. This cocktail of circumstances ultimately results in the affected individual taking heightened employment risks, accepting a job that is ‘too good to be true’ and succumbing to deception and exploitation in the process. This meets the definition of human trafficking—a sequence of events can be traced back to the initial impact of a disaster. The notion of susceptibility to trafficking building up over several years can also tie into Chambers' (1983, p. 112) ‘ratchet effect’ theory, whereby each disaster makes a population more vulnerable than the last.

**FIGURE 2:**
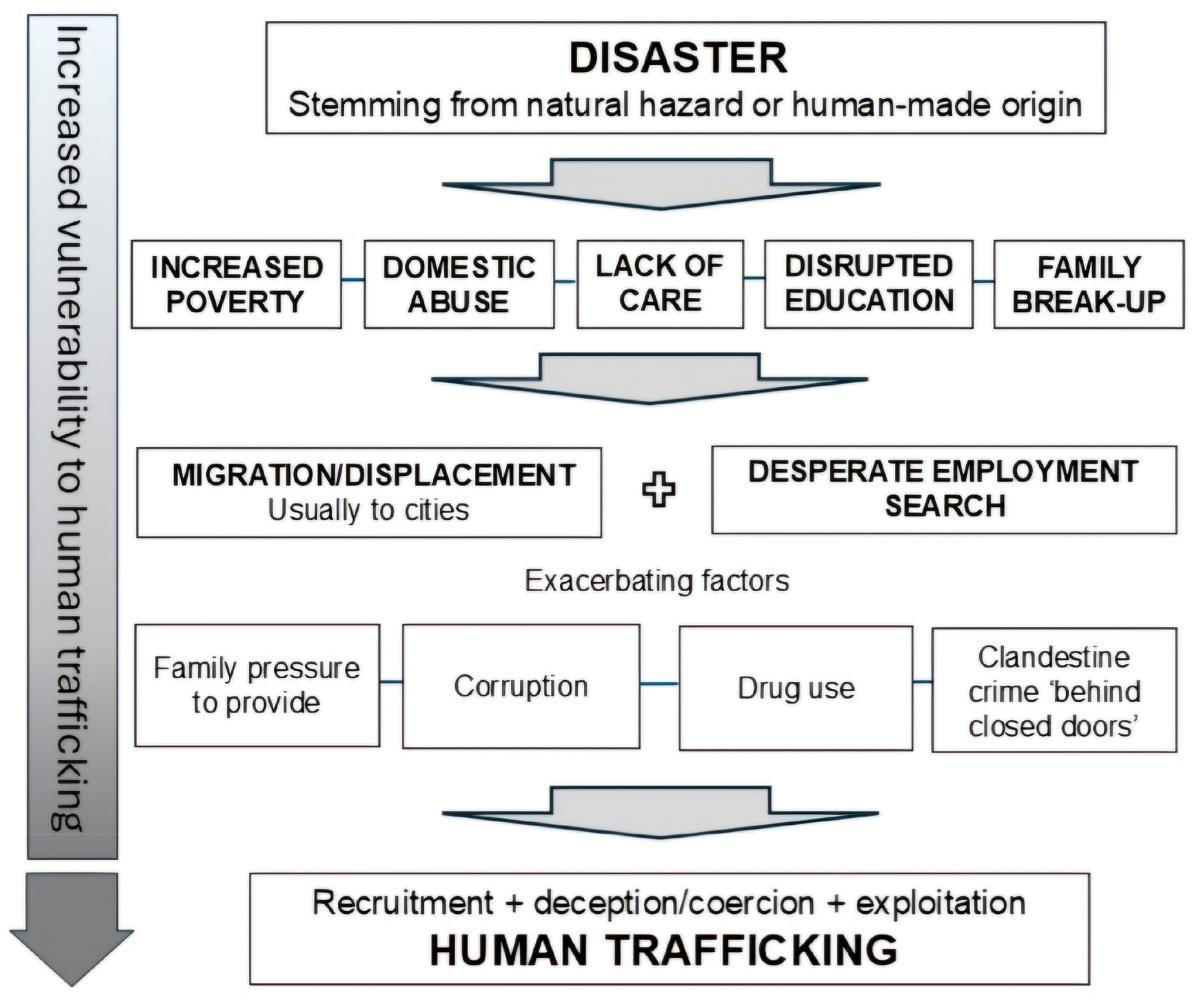
Disaster–trafficking sequence as presented by trafficking survivors in this study. **Source:** author.

This can be termed the ‘slow‐burn effect’. In other words, a disaster can spark a chain of events, the impact of which mounts up over several years, or, in its most pronounced form, even has an effect in childhood, which leads to trafficking later in life. This chain of events is presented visually in Figure 2.

While all of these scenarios are to some extent referenced theoretically in the existing literature, there is little or no narrative surrounding how they unfold in lived experience—nor regarding the significant passage of time between a disaster and the moment of human trafficking. With reference to the ‘slow‐burn effect’, there appears to be no reference to this concept in the academic literature, although a handful of NGO reports do allude to the phenomenon. First, a 2016 report by the IOM notes that, while most disaster–trafficking claims appear to focus on rapid‐onset events which cause immediate devastation, the cumulative effect of lower‐level, repeated disasters such as annual flooding or drought should not be overlooked (Coelho et al., 2016, p. 3). In addition, an excellent and wide‐ranging NGO report from the Philippines states that the disaster–trafficking link, while evident, is not direct (Alburo‐Cañete et al., 2014, p. 193). The researchers, as part of a three‐year project with a consortium of three locally‐based NGOs, interviewed 88 respondents who had either experienced trafficking themselves, or knew a person close to them who had been trafficked. They conclude that ‘there is a clear link between vulnerabilities to disasters and human trafficking’ but that this relationship is indirect (Alburo‐Cañete et al., 2014, p. 1). They point out that disaster can have a direct impact on livelihoods, which, in turn, leads to coping strategies that may later result in human trafficking (Alburo‐Cañete et al., 2014, p. 194). Lastly, a 2015 Master's thesis highlights that, theoretically in the context of disaster–trafficking, the impacts of a calamity ‘may be felt not only in the immediate aftermath of a shock, but still months and years later’ (Brülisauer, 2015, p. 2).

## 8 POLICY RECOMMENDATIONS

The primary recommendation emerging from this study is for further empirical research on where, when, how, and for whom vulnerability to human trafficking is increased. Rather than focusing on the assumption that risks escalate in a disaster's immediate aftermath, policymakers could cast the net wider and consider how disasters can set off a chain of events which result in trafficking over a longer period of time. This knowledge, if further developed, could play into targeted awareness campaigns, education, and robust and consistent legislation, as well as the enforcement of those laws.

Disaster preparedness, such as better housing and building away from flood‐affected areas, could also have prevented the chain of events that led to some of the trafficking cases in this study, although this is arguable and cannot be said with certainty.

Such policy recommendations are approached with caution, noting that ‘many Western academics who “collaborate” with local researchers use their presumed superiority. .. to “remake” these people and places. They would replace old, dysfunctional ways of being with “smarter” ones, offering “innovations” that, although often unintentional, nevertheless eliminate public goods, permanently displace vulnerable residents, and devastate social infrastructure’ (Chmutina et al., 2025, p. 11).

The final point to make is that, as I can attest, the opportunity to hear directly from those who have been trafficked is rare. Thus, well‐trained individuals in trusted positions who already have this access could, in addition to their regular and significant workload, collect and disseminate appropriate, anonymised, first‐hand testimony.

## 9 CONCLUSION

This empirical study endorses theoretical assertions in the literature of a link between disaster and human trafficking. Yet, testimony presented by trafficking survivors is a far cry from the caricature of shadowy figures descending on to the streets of a natural hazard‐battered city to recruit their victims, with weakened law enforcement unable to intervene in the post‐disaster mayhem. Rather, survivors explained how disaster can create a ‘shock’ at a certain point in life which sparks a chain of events that, ultimately, may result in trafficking, although often not until many years later. This can be termed the ‘slow‐burn effect’ because of its protracted nature.

Each story is, of course, completely unique and the extent to which disaster was a catalyst for trafficking appears to vary significantly between respondents. At one end of the spectrum, disaster is merely a single factor among many that create a ‘tipping point’, prompting economic desperation and a search for work which morphs into trafficking. At the other end, four individuals referred to distinct and significant disasters that had a sudden and devastating impact on their family's economic situation. This, in turn, forced the individuals to seek work urgently, resulting in human trafficking. While other variables were at play, disaster was therefore perceived by a small number of respondents to be a central feature of their trafficking journey.

Until now, the disaster–trafficking link was presented theoretically in the literature, with an apparent void of evidence from trafficking survivors themselves. This study begins to fill that empirical vacuum and add much‐needed nuance and colour to existing theory. Survivors' perception of disaster as a trafficking catalyst is arguably a strong starting point to steer current narratives in the extant literature.

With a rising global population, climate change, and natural hazards becoming ‘more intense and more likely’ (World Weather Attribution, 2023), this area of research also has important implications for the future. It makes the need for knowledge even more pressing as policymakers seek to empower and protect those affected by natural hazards around the globe. Understanding where and when the risks of post‐disaster human trafficking are likely to occur, and who is going to confront them, will prove a useful tool in this endeavour.

## ETHICS STATEMENT

All of the respondents in this study had been subjected to exploitation. It was established before the research began that all of them had exited the trafficking situation and were in no danger from the perpetrators. All trafficking survivors were aged 18 or over, and a trained social worker was present throughout the research. Group debrief sessions were held with respondents after each set of interviews. The ethics of data collection and analysis were approved in a two‐stage ethics review at SOAS University of London in summer 2016.

## Data Availability

The data that support the findings of this study are available on request from the corresponding author. The data are not publicly available due to privacy or ethical restrictions.

## Endnote

^1^
The full text of Article 3 of the Protocol to Prevent, Suppress and Punish Trafficking in Persons Especially Women and Children, supplementing the United Nations Convention against Transnational Organized Crime (also known as the Palermo Protocol) is as follows: ‘(a) “Trafficking in persons” shall mean the recruitment, transportation, transfer, harbouring or receipt of persons, by means of the threat or use of force or other forms of coercion, of abduction, of fraud, of deception, of the abuse of power or of a position of vulnerability or of the giving or receiving of payments or benefits to achieve the consent of a person having control over another person, for the purpose of exploitation. Exploitation shall include, at a minimum, the exploitation of the prostitution of others or other forms of sexual exploitation, forced labour or services, slavery or practices similar to slavery, servitude or the removal of organs; (b) The consent of a victim of trafficking in persons to the intended exploitation set forth in subparagraph (a) of this article shall be irrelevant where any of the means set forth in subparagraph (a) have been used; (c) The recruitment, transportation, transfer, harbouring or receipt of a child for the purpose of exploitation shall be considered “trafficking in persons” even if this does not involve any of the means set forth in subparagraph (a) of this article; (d) “Child” shall mean any person under eighteen years of age’. For more information, see https://www.ohchr.org/en/instruments-mechanisms/instruments/protocol-prevent-suppress-and-punish-trafficking-persons (last accessed on 14 March 2025).
